# Comparative genome and transcriptome analysis of diatom, *Skeletonema costatum*, reveals evolution of genes for harmful algal bloom

**DOI:** 10.1186/s12864-018-5144-5

**Published:** 2018-10-22

**Authors:** Atsushi Ogura, Yuki Akizuki, Hiroaki Imoda, Katsuhiko Mineta, Takashi Gojobori, Satoshi Nagai

**Affiliations:** 1 Nagahama Institute of Bioscience and Technology, 1266 Tamura, Nagahama, Shiga 5260829 Japan; 2 National Research Institute of Fisheries Science, 2-12-4 Fukuura, Kanazawa, Yokohama, Kanagawa 236-8648 Japan; 3 Computational Bioscience Research Center (CBRC), King Abdullah University of Science and Technology (KAUST), Thuwal, 23955-6900 Saudi Arabia

**Keywords:** Genome, Transcriptome, Red tide, Harmful algal bloom, Oxidative stress response, Response to cytokinin

## Abstract

**Background:**

Diatoms play a great role in carbon fixation with about 20% of the whole fixation in the world. However, harmful algal bloom as known as red tide is a major problem in environment and fishery industry. Even though intensive studies have been conducted so far, the molecular mechanism behind harmful algal bloom was not fully understood. There are two major diatoms have been sequenced, but more diatoms should be examined at the whole genome level, and evolutionary genome studies were required to understand the landscape of molecular mechanism of the harmful algal bloom.

**Results:**

Here we sequenced the genome of *Skeletonema costatum*, which is the dominant diatom in Japan causing a harmful algal bloom, and also performed RNA-sequencing analysis for conditions where harmful algal blooms often occur. As results, we found that both evolutionary genomic and comparative transcriptomic studies revealed genes for oxidative stress response and response to cytokinin is a key for the proliferation of the diatom.

**Conclusions:**

Diatoms causing harmful algal blooms have gained multi-copy of genes related to oxidative stress response and response to cytokinin and obtained an ability to intensive gene expression at the blooms.

**Electronic supplementary material:**

The online version of this article (10.1186/s12864-018-5144-5) contains supplementary material, which is available to authorized users.

## Background

Diatoms are a unicellular, diploid, photosynthetic, eukaryotic microalgae, which distribute throughout marine and freshwater systems [1], and the group exists in a huge variety of shapes and sizes. They contain tens of thousands of species [2], even on conservative estimates, showing their explosive diversification, yet they have appeared only since the early Mesozoic period [3]. As a unique feature of diatom cells, they are enclosed by a cell wall made of silica, called a frustule, and their vegetative cells reproduce by asexual cell division. However, cell size decreases as a result of the mechanism of frustule formation. The valve diameter linearly decreases with the number of cell divisions, and the correlation is termed the “McDonald and Pfitzer’s rule” [4]. When the cell size decreases markedly, the cells become unable to divide further and die [5, 6]. Consequently, they must restore their cell size by auxospore formation (sexual processes in the strict sense of the word) and vegetative cell enlargement (pseudo-auxospore formation, i.e., an asexual process) [1]. Diatoms are the most dominant microalgal group in coastal waters and often form dense blooms sporadically [7, 8]. The primary use for silicic acid is in the construction of their frustules [9]. Therefore, the biogeochemical cycle of silicon is dominated by the activity of the diatoms in marine systems [10], and it has been estimated that globally, the diatoms uptake and process 240 Tmol Si per year. There is an estimate for global net primary production (NPP) of 104.9 petagrams of carbon per year, of which 46% is oceanic and 54% is terrestrial [11]. Diatoms could account for between 40 and 45% of oceanic production, indicating 20 petagrams of carbon fixation per year. This carbon fixation indicates that diatoms are remarkably abundant, playing an essential part in the global cycling of many elements, but particularly C and Si [12].

*Skeletonema costatum* (Greville) Cleve is considered to be one of the most abundant and cosmopolitan diatoms in the coastal marine phytoplankton. It also features prominently as a key organism in research fields ranging from biochemistry, ecophysiology, and molecular biology to ecology, oceanography, and aquaculture [13]. Recent studies utilizing electron microscopy and large subunit rDNA sequences from marine strains revealed that *S. costatum* sensu *lato* (s.l.) consists of a series of genetically and morphologically distinct species [13–16]. At present, eleven species belong to this genus [17]. However, most reports from coastal Japan described blooms of the species *S. costatum*, which was the first described in the genus, and until recently it was believed that only *S. costatum* and *S. tropicum* appear in Japanese coastal waters. Diatoms have been the most dominant phytoplankton group (> 90%) over a 35-year period, and the genera *Skeletonema* and *Chaetoceros* are two major diatom groups in Japanese waters [9].

On the contrary to the great role for the carbon fixation by diatoms, red tides or harmful algal blooms caused by diatoms have been a major problem for the environment and the fishery industry. The red tide is named after the color of the water, which changes to reddish brown, depending on the pigment of plankton. Harmful algal bloom represents the same phenomena as the red tide and widely used in the environmental problem. Harmful algal blooms are known to be caused by changes in nutritional conditions, temperature, but they do not necessarily occur in the same situation. Recent studies also suggested that some viruses and bacterial interactions might be the causal factor, but details are not yet clarified [18]. Harmful algal blooms also have adverse effects, such as the suffocation of fish, due to the lowering of the oxygen concentration in the surrounding marine environment or due to the toxins produced by diatoms. Species of the *Skeletonema* are harmful and cause a severe economic loss in Japanese aquaculture because they utilize nutrients necessary for the growth of the red algae *Porphyra* (nori) in winter [19]. A recent study revealed that *S. costatum* sensu stricto, *S. dornii*, and *S. japonicum* distribute widely and abundantly in the western part of Japan ([20, 21], Nagai unpublished). The shell of this diatom is a cylinder, about 22 μm in diameter, and is common in the bay and coastal waters. The ecological importance and the complicated speciation process of the genus *Skeletonema* motivated us to start the complete genome analysis in these *Skeletonema* species. Thus, it is of importance to reveal the genetic background of diatoms and the molecular mechanism of the proliferation of diatoms as the molecular mechanism of harmful algal blooms is still unclear.

In diatoms, the complete genome sequences were reported only on two diatoms, *Thalassiosira pseudonana* (Tp) as the representative of the marine centric diatom and *Phaeodactylum tricornutum* (Pt) as the representative of the pennate diatom [22, 23]. The dominant species of harmful algal blooms in Japan is the diatom, *Skeletonema costatum* (Sc). Although we know about the characteristics of this diatom, we do not know the whole genome. In this study, we sequenced the whole genome of Sc, and performed a comparative genomic analysis of the constituent species causing harmful algal bloom and out-group, *Vitrella brassicaformis* (Vb), which is a single-cell organism belonging to chromomers that are close to Stramenopile involving Sc, Tp, and Pt [24]. Vb is also known as the species that does not cause harmful algal bloom, so that this species is useful not only for the outgroup of evolutionary studies but also for screening genes responsible for harmful algal bloom. The phylogenetic relation of Vb, Tp, and Pt used for analysis is already known in the publication of Vb [23]. We also performed RNA-sequencing of Sc under various conditions by changing temperatures, light, and nutrition. By conducting transcriptome analysis, we analyzed changes in gene expression corresponding to the changes of conditions to elucidate the molecular mechanism of harmful algal bloom.

## Results

### Evaluation of genome assemblies and gene models

**Fig. 1:**
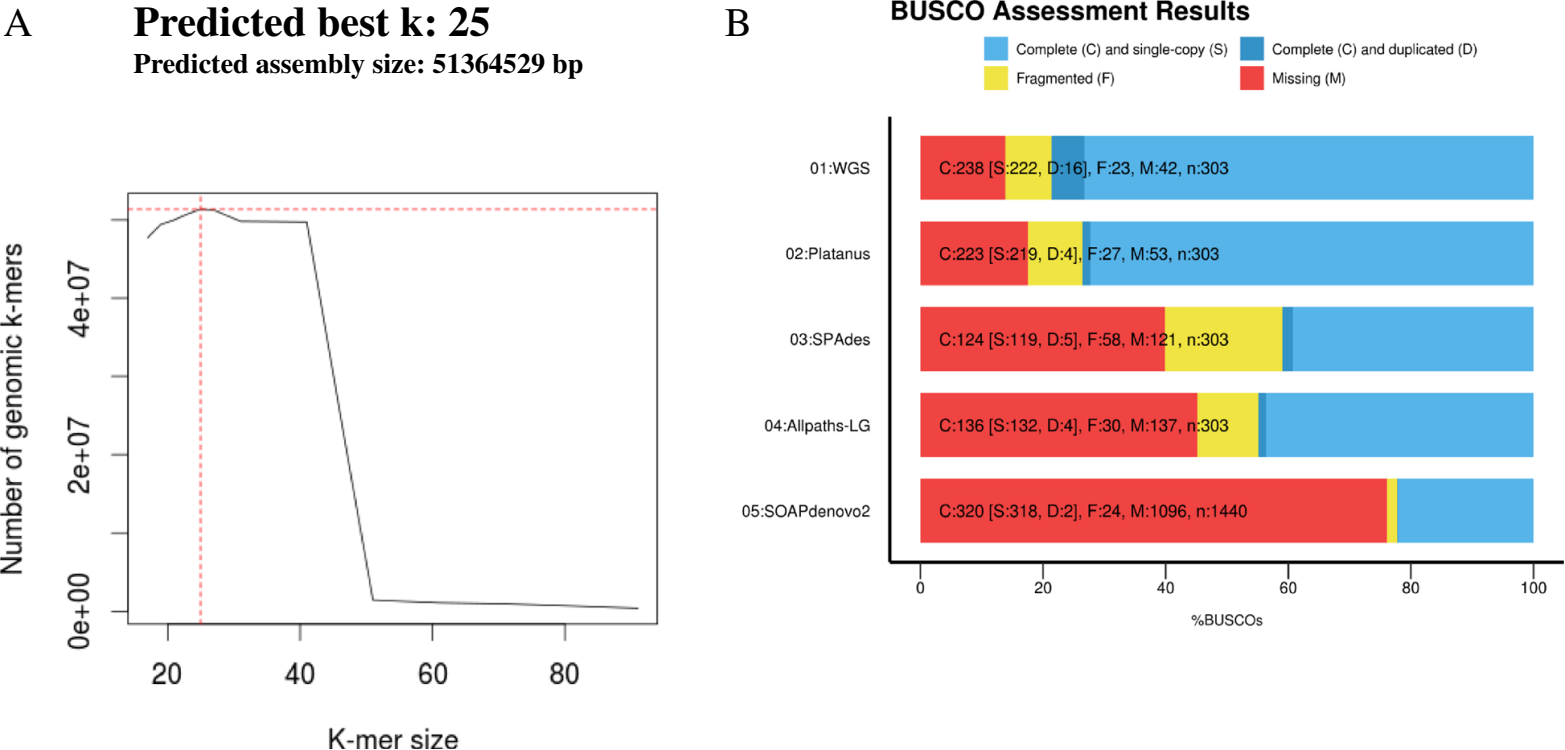
Genome size estimation by k-mer analysis and the common eukaryotic genes conservation rate. K-mer analysis represents best k-mer as 25-mer and the estimated genome size is 51,364,529 bp (**a**). BUSCO analysis represents conservation rate of the common eukaryotic genes for four different assemblies. Color key is described in the figure (**b**)

To obtain genome and gene model of Sc with higher quality, we tried to use four different genome assembly software, WGS, Platanus, Allpaths-LG, SOAPdenovo2, and SPAdes. We compared the assembly results with others from the viewpoint of genome size, the number of contigs, the largest contig, total length, GC content, N50, BUSCO assessment for the common eukaryotic genes conservation rate (ECR). As a result, the assembly by Platanus showed reasonably better results than others on the basis of the number of contigs, larger N50, close value to the estimated genome size, and ECR (Fig. 1, Table 2). In detail, we have conducted genome size estimation using k-mer analysis to assess differences between estimated genome size and assembled genome sizes (Fig. 1). From the k-mer analysis, genome size of Sc is estimated as 51,364,529 bp and Platanus (46.9 Mb) and SOAPdenovo2 (52.7 Mb) showed close genome size. For the genome quality in terms of gene model estimation we have performed BUSCO analysis, which assesses genome assembly with benchmarking universal single-copy orthologs. From this analysis, WGS (238 complete genes) and Platanus (223 complete genes) showed higher quality than others. WGS contains 16 duplicate genes that might be derived from redundant genome assembly as the assembled genome size by WGS is much larger than estimated from k-mer analysis. Taken together, we conclude that genome assembly by Platanus would be adopted. We then compared the genome and gene model of Sc with other diatoms and Vb, and it was suggested that Sc had larger genome size than other diatoms, and the number of genes was also larger than that of other diatoms (Additional file 1: Table S1). The genome size of Sc was larger than other diatoms, so that we checked if the reason for the larger-sized genome is due to repeat sequences. We searched repeat sequences by RepeatMasker and found that Sc possessed many number of simple repeats comparing with other diatoms, but possessed less SINE and LINE (Additional file 1: Table S1, sheet1-Repetitive element).

### Orthologous analysis of common genes in harmful algal bloom causing diatom and Sc-specific genes

**Fig. 2:**
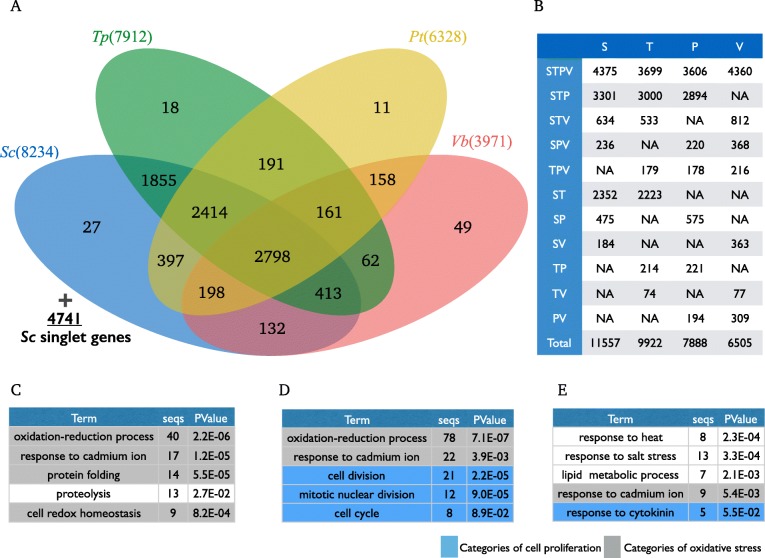
Orthologous analysis and Gene set enrichment analysis of common genes of harmful algal bloom, Sc-specific genes, and Sc-duplicated genes. **a** Venn diagram of sharing pattern of orthologous gene groups. The digit indicates the number of orthologous gene groups. Sc singlet genes represents the number of non-orthologous genes in Sc, and passed to the list of Sc-specific genes. **b** The number of genes in the orthologous gene groups. First column represents conservation pattern of orthologous genes in S-Sc, T-Tp, P-Pt, V-Vb. For example, STPV has 2798 orthologous group as shown in the panel A, and includes 4375 genes in Sc, 3699 genes in Pt. **c** Gene set enrichment analysis of the common genes for the harmful algal bloom. **d** Gene set enrichment analysis of Sc-specific genes. **e** Gene set enrichment analysis of Sc-duplicated genes

To clarify the common genetic background in the diatom and to specify the genetic novelty of Sc from other diatoms, we estimated orthologous gene groups that share the same common ancestral gene and are supposed to have the same functionality. The orthologous gene groups conserved only in the diatom but not in other species, Vb in this case, might have relationships with the molecular mechanism of the harmful algal bloom. On the other hand, genes that are only found in Sc but have not in other species could be regarded as Sc-specific genes, which might be related to the functional novelty in Sc. We therefore conducted orthologous gene groups estimation analysis using OrthoFinder and classified orthologous gene groups by conservation patterns that were shown in the Venn diagram (Fig. 2a). As one orthologous gene groups could contain more than one genes, the number of genes and the number of the orthologous gene groups was different, which were shown in the panel B (Fig. 2b, Additional file 1: Table S1, sheet2-#genes in the venn diagram). Total number of genes, 16,449 genes can be categorized to 11,577 genes (in Orthologous groups), 4741 (in singlet genes), and 151 genes (from 27 Sc only orhologous groups). The proportion of conserved genes between Sc and Tp that are closest to Sc were about 65.4%, which were a total of Sc-Tp shared genes (4375-STPV, 3301-STP, 634-STV, and 2352-ST) divided by the total number of Sc genes (11,557 + 4741). In the same manner, the number of conserved genes among the species tested could be calculated from the digit in the same figure and table. Diatom common genes were preserved about 45% from the number of STPV and STP genes that may have the essential role in the functionality of the harmful algal bloom. On the contrary to the conserved genes among species, there are genes only found in Sc. Such Sc-specific genes can be categorized to two types, duplicated and singlet genes. The former can be detected as 27 Sc-specific orthologous groups that consist of 151 genes, and the latter can be detected as genes without any homology to other species, 4741 Sc-singlet genes, which genes might be important for functional novelty in Sc.

### Enrichment analysis for the common genes for the harmful algal bloom and Sc-specific genes, Sc-duplicated genes

We conducted gene set enrichment analysis to clarify what functional categories are comprised of the Sc-specific genes and the common diatom genes related to the harmful algal bloom. To perform gene set enrichment analysis, GO annotation is required, so we first conducted blast search against the DB of *Arabidopsis thaliana*, which is the model organism with annotation for gene ontology categories. As a result, the oxidation-reduction process and response to cadmium ion were shown from the common genes for the harmful algal bloom, the Sc-specific genes, and the Sc-duplicated genes (Fig. 2c, d, e, Additional file 1: Table S1, sheet3-Duplication_list). In the analysis, the function related to oxidative stress was significantly enriched in the common genes for the harmful algal bloom and the Sc-specific genes. Cell division and Mitotic nuclear division were also shown from the common genes for harmful algal bloom. The genes that related to proliferation were significantly enriched in the orthologous gene group found in the red tide causing diatoms. These indicates that, in the process of algal bloom, massive photosynthesis occurs and diatoms should response to this stress by duplicating genes related to oxidative stress. Genes related to cell proliferation such as cell division and mitotic nuclear division seem also important to fit rapid proliferation during algal blooms.

### Transcriptome analysis

To elucidate the molecular mechanism of the harmful algal bloom, it is necessary to analyze what kind of fluctuation of Sc genes occurs under the harmful algal bloom condition. For this purpose, we performed a transcriptome analysis that could comprehensively analyze the expression status of genes in various conditions imitated for the harmful algal bloom causing situations. For the culture conditions for this experiment, we changed temperatures, light, and nutrition to test which genes are responsible for the environmental changes.

**Fig. 3:**
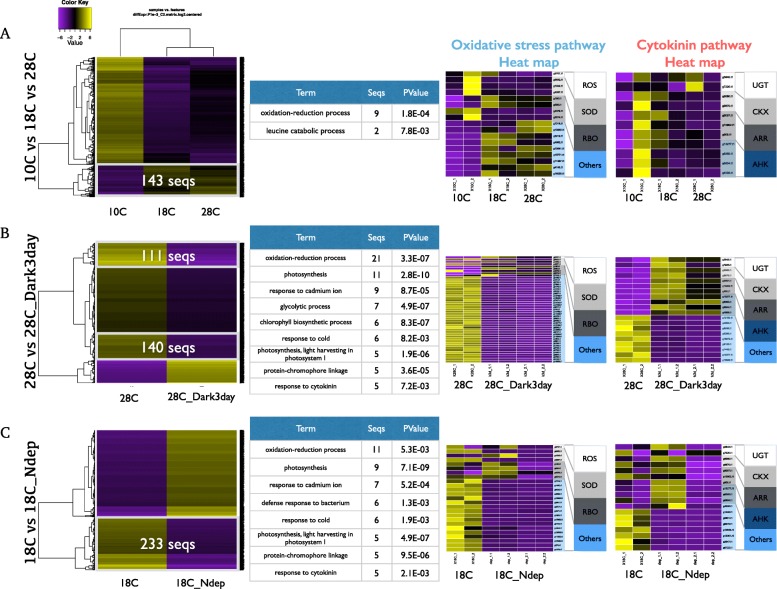
Differentially expressed genes extracted from RNA-seq data under different conditions. **a** Differentially expressed genes among Sc culture samples for different temperatures, 10 °C, 18 °C, and 28 °C. 143 genes were extracted as highly expressed genes at high temperatures with FC > 2. These 143 genes were then used in the gene set enrichment analysis. As oxidative stress pathway was enriched, gene expression intensities of relative genes were extracted and used in the heatmap shown in the right-hand side. **b** Differentially expressed genes among Sc culture samples for different light conditions. 251 genes were extracted as highly expressed genes at lighter condition with FC > 2. Following steps are the same as above. **c** Differentially expressed genes among Sc culture samples for different nutrient conditions. 233 genes were extracted as highly expressed genes at abundant nitrogen condition. Following steps are the same as above

First, we changed the temperature condition from cold (10 °C) to normal (18 °C), and an even higher temperature (28 °C) where the red tide is likely to occur. Then, highly and differentially expressed genes were extracted, and we demonstrated the heat map on the expression level of Sc under the conditions of 10, 18, and 28 °C (Fig. 3a). By the gene set enrichment analysis, the expression was elevated under the red tide conditions (Fig. 3b, c). As a result, when comparing 18 °C and 28 °C where the red tide is likely to occur, 143 genes were highly expressed at 28 °C (Fig. 3a). Enrichment analysis performed on the identified genes indicated that the category, “oxidation-reduction process,” (related to oxidative stress) was significantly highlighted.

Next, we changed the light condition (28 °C) where the red tide was likely to occur. Differentially and highly expressed genes were extracted, and enrichment analysis was performed to identify functional categories related to the red tide. A heat map on the expression level of Sc under the conditions of light and darkness representing 251 genes was identified (Fig. 3b). Enrichment analysis of these 251 genes was performed, indicating that the oxidation-reduction process including some other photosynthesis related process, and response to cytokinin were significantly enriched. The genes for oxidation-reduction process were also involced in the category of photosynthesis, response to cadmium ion, glycolytic process, chlorophyll biosynthesis process. Response to cytokinin is a key to the growth of plants. We therefore investigated gene expression profiles of oxidative stress pathway and cytokinin pathway including non differentially expressed genes.

Finally, we changed the nutritional conditions and extracted highly and differentially expressed genes in good condition for the red tide. A heat map on the expression level of Sc under poor nitrogen conditions and normal conditions were shown. As a result, 233 sequences were identified for the conditions where the red tide was likely to occur (Fig. 3c). Enrichment analysis of these 233 genes showed that the oxidation-reduction process including some other photosynthesis related process response to cadmium ion, and response to cytokinin were significantly enriched again. Even though cadmium is very toxic to organisms, it is reported that cadmium enhances the growth of the marine diatom under conditions of zinc limitation [25, 26].

From the transcriptome analysis, genes related to oxidative stress were significantly enriched under temperature, light, and nutrient conditions in which the red tide is likely to occur as the same as genome analysis. These genes were associated with the proliferation of cells and related to the harmful algal bloom.

### Comparison of the results from transcriptome and genome analysis

**Fig. 4:**
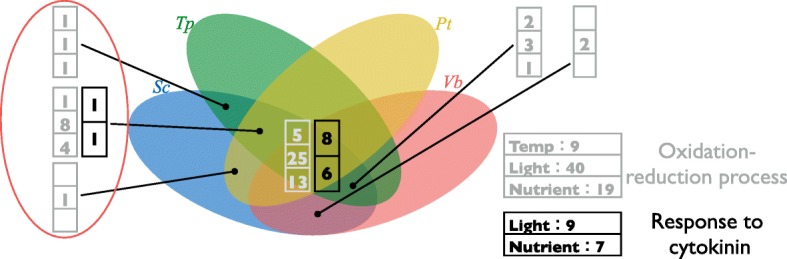
Differentially expressed genes related to Oxidation reduction process and Response to cytokinin on the venn diagram. Grey rectangle represents the number of differentially expressed genes under different temperatures (up), different light conditions (middle), and different nutrient condition (bottom). Black rectangle represents the number of differentially expressed genes under different temperatures (up), different light conditions (middle), and different nutrient condition (bottom)

When comparing the results from the genome and transcriptome analysis, the same category of genes, response to the oxidative stress and response to cytokinin seemed to be enriched in both results. To examine this observation, sharing pattern of the common genes of the harmful algal bloom and SC-specific genes, and differentially expressed genes in the three different conditions were merged into the same Venn diagram. As a result, many of Oxidation-reduction process, Response to cytokinin belonged to the common group of algae (Sc, Tp, Pt, Vb) (Fig. 4).

### Gene expression level of oxidative stress pathway and cytokinin pathway

**Fig. 5:**
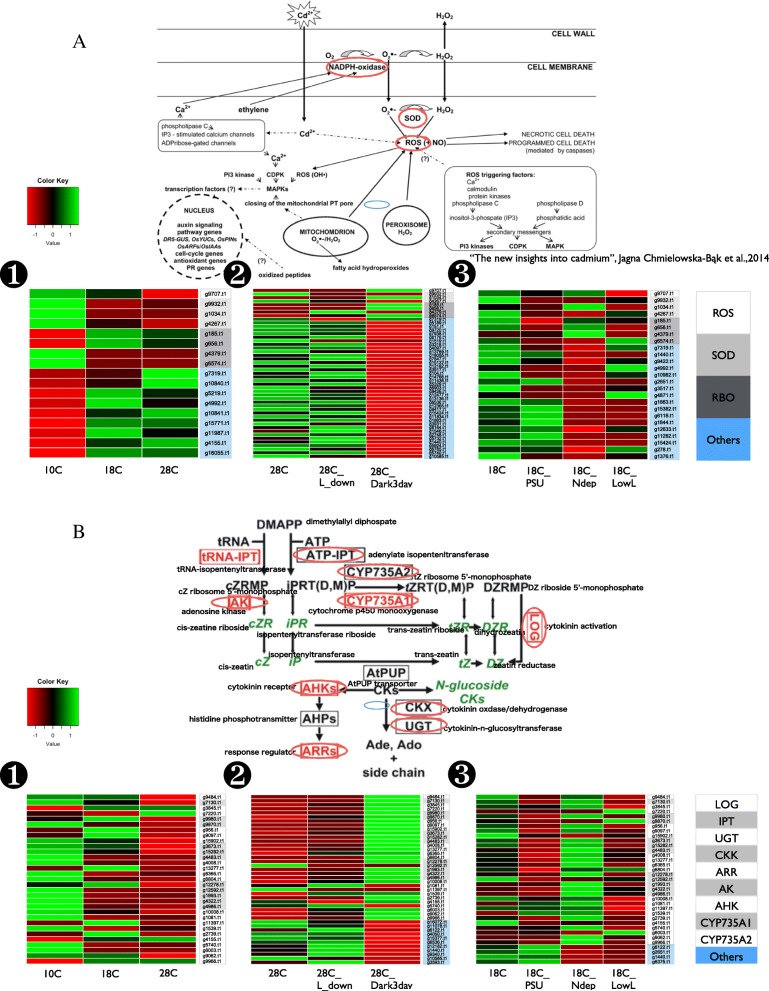
Detailed analysis of gene expression differences under different conditions on oxidation reduction pathway (**a**) and the response to cytokinin process (**b**). Red to green color represents log gene expression of ROS, SOD, RBO, and other genes involved in the pathway (**a**), and LOG, IPT, UGT, CKX, ARR, AK, AHK, CYP735A1, CYP735A2, and other genes involved in the pathway (**b**). Pathways was modified from the figures in “The new insights into cadmium” (Jagna Chmielowska-Bąk et al.,2014), and “Antagonistic roles of abscisic acid and cytokinin” (Yandu Lu et al.,2014), respectively

In the transcriptome study, we observed significant enrichment of genes related to oxidative stress in the different conditions. We, therefore, checked gene expression differences in the pathway of response to the oxidative stress to examine this finding is not affected by the limited number of genes but the whole pathway (Fig. 5a). As a result, in the 28-degree condition from two different sample sets for temperatures and lights, up-regulation of oxidative stress pathway was observed compared with lower temperatures. In the different nutrients condition, down-regulation of oxidative stress pathway was observed in N-depletion and lower irradiance. We also checked gene expression differences in the pathway of cytokinin, which is essential for the growth in plants (Fig. 5b). There are no strong correlation in the different temperatures and different nutrients, but down-regulation of cytokinin pathway was observed in dark conditions.

### Silicate-related genes

Diatom utilize silicic acid from seawater by silicon transporter (SIT genes) and they construct cell walls using silaffin genes. Based on the estimation of silicate and silaffin genes in the four species (Additional file 1: Table S1, sheet4-#silicate & silaffin), Tp possessed higher number of genes comparing with other species, whereas Sc has also high number of silicate-related genes. As homology search were performed using query sequences from Tp, the number of genes of other than Tp might be underestimated. However, these genetic diversities of silicate-related genes might reflect the complexity of diatom structures. Regarding Pt, it is known that the putamen is not formed much compared with other diatoms, consequently less number of silicate-related genes seemed reasonable.

## Discussion

The whole genome of *Skeletonema costatum*, Sc was determined by Illumina Hiseq 2000, the next generation sequencer. The genome size of Sc was 46.9 Mb, and the number of genes was 16,449, both of which were larger than other diatoms. The completed Pt genome is approximately 27.4 megabases (Mb) in size, which is slightly smaller than Tp (32.4 Mb), and *P. tricornutum* is predicted to contain fewer genes (10,402 as opposed to 11,776, Table 1). Despite the fact that the pennate and centric lineages have only been diverging for 90 million years, their genome structures are dramatically different, and a substantial fraction of genes (40%) are not common in these representatives of the two lineages. Evolutionary analysis of molecular divergence compared among Tp, *Arabidopsis* and *Chlamydomonas* reveals their rapid diversifications of genes in diatoms. As shown in Table 2, the genome of Sc has many contigs, and a large number of fragment sequences are generated, which might be caused by fragmentations of contigs and over-estimation of genes. In the orthologous gene analysis, we estimated orthologous gene groups using similar length of genes, representing reliable orthologous gene sets. For Sc, many of the putamen-related genes were found since it is the closest to Tp among the three species. Growth-related genes, such as cytokinin-related genes were also found in the common genes for the red tide from orthologs in four species (Figs. 3 and 4). Furthermore, oxidative stress-related genes were found in Sc-specific genes; the common genes for the red tide. Transcriptome analysis revealed that oxidative stress-related genes and cytokinin-related genes were highly expressed with the condition preferable for the red tide, as compared with the condition for non-red tide (temperature, light, and nutritional conditions). Cytokinin has been shown to be involved in cell proliferation in other algae. These findings from both genome analysis and transcriptome analysis are consistent each other, indicating that the evolution of growth-related genes gained in the genome by the duplication might be relevant for their higher expression required for the process of the red tide. On the other hand, Superoxide dismutase (SOD) that catalyzes the dismutation of the superoxide radical into oxygen was works actively in the red-tide condition. This is also consistent with the fact that one of the key genes for the pathway responding to the oxidative stress, ROS, was activated by the light condition.

**Table 1:** Comparison of genome assembly results of Sc using four different assemblers

	Genome size	#Scaffolds	Longest scaffold	Average length	N50
WGS	60.2 M	38.8 K	89.1 K	1.6 K	4.7 K
Platanus	46.9 M	62.1 K	122.6 K	0.8 K	24 K
Allpaths-LG	29.7 M	6.2 K	4.8 K	4.8 K	8.6 K
SOAPdenovo2	52.7 M	150.5 K	0.4 K	0.4 K	0.8 K

**Table 2:** Comparison of genome assemblies of 4 species tested

	Sc	Tp	Pt	Vb
Genome				
Genome size	46.9 Mb	34.5 Mb	31.0 Mb	72.7 Mb
Total reads	6.3G	440 M	322 M	–
N50	24.2 K	7 K	25 K	148 K
GC content	45.1%	46.9%	48.9%	58.1%
Gene model				
Total gene number	16,449	11,390	10,025	23,034
Average length	1.1 K	2.0 K	1.5 K	1.1 K

## Conclusions

*Skeletonema costatum* gained duplicated genes of growth related genes such as cytokinin for rapid growth for algal bloom, by which the diatom can proliferate when the environmental condition matched to their preferences. In the bloom condition, they should adapt to higher oxidative stress condition, so that they can overcome this condition by higher activity of oxidative stress response.

## Methods

### *S.costatum* sample acquisition and extraction of genomic DNA

An axenic and clonal strain of *Skeletonema costatum* (Ariake8) was isolated from a bloom in Ariake Sea (33o06.31’N; 130o22.15E) in April 2013 by micropipetting single chains. f/2 medium was modified by addition of 10 μM of selenious acid (H2SeO3) and without copper sulfate hydrate in the stock solution of the metal mixture (Nagai et al. 2004). The clonal strain was maintained in 20 ml of f/2 medium based on enrichment of natural seawater collected from Tokyo Bay (salinity adjusted to 30 psu) in a 75 mL capacity plastic tube at a temperature of 18 °C under an irradiance of 100 μmol m-2 s-1 provided by cool-white fluorescent lamps with a 12:12 h L:D cycle. For the whole genome analysis, the strain was incubated in 3 × 400 mL of the modified f/2 medium under the same conditions for the maintenance culture for one week, and the vegetative cells were harvested by filtrating through 1-μm-pore-size polycarbonate filters (Nuclepore membrane, GE Healthcare, Tokyo, Japan). Genomic DNA was extracted from the harvested cells on the filter with a modified SDS-Proteinase K method (TE buffer: 10% SDS: 20 mg mL-1 Proteinase K (Qiagen) = 16:15:1).

### Preparation of transcriptome samples

For the transcriptome analysis, the effects of incubation temperatures, lights, and nutrients (nitrogen depletion) on the gene expression of *Skeletonema* were studied. As for the temperature (experiment-1), the strain was incubated at 400 mL of the modified f/2 medium under the same conditions with the maintenance cultures, i.e. an irradiance of 100 μmol m^− 2^ s^− 1^ with a 12:12 h L:D cycle, at three different temperatures of 10, 18 and 28 °C during their exponential growth phases (4–7 days). 2) As for the irradiance, the strains were incubated at the same conditions of the maintenance cultures except for the irradiance (20 μmol m^− 2^ s^− 1^) for 7 days (experiment 2–2). Similarly, the strain was incubated under the same conditions with the experiment-1 at 28 °C for 4 days and put the culture in the dark for 3 days (experiment 2–2). The strain was also incubated under the same conditions with the experiment-1 at 28 °C for 4 days, then it was put at a lower irradiance of 20 μmol m^− 2^ s^− 1^ for 24 h (experiment 2–3). As for the nutrients, the strains were incubated at the same conditions of the experiment-1 at 18 °C without addition of nitrogen (without of 882.4 μM as NaNO3) for 7 days. At the end of the incubation the cultures were harvested on the same filter used in the whole genome analysis, and the total RNA was immediately extracted by TRIzol Plus RNA Purification Kit (Life technology). We conducted transcriptome analysis with two biological replicates.

### Genome sequencing and RNA sequencing

Sequencing was performed using the Illumina Hiseq 2000, the next-generation sequencer. For genome sequencing, library construction of Pair End libraries with insert lengths of 200 bp, 500 bp, and a Mate-pair library with an insert length of 2000 bp were performed by the default protocol and these libraries were used for sequencing. We obtained a total of 6.3Gbps from gDNA of *S. costatum* and used them for de novo assembly. RNA-seq libraries were prepared for sequencing with Illumina Hiseq 2000. These sequence raw reads were deposited to DDBJ DRA sequence read archive (ID:DRA007346).

### Quality control and genome assembly

Data was trimmed by default with the Solexa QA (v3.1.7.1) [27], which is quality control software that eliminates unreliable parts of sequences extracted by the Illumina Hiseq 2000. Genomic assemblies were performed using the data after trimming. To generate a more accurate Sc. genome, we evaluated the assembled genomes by WGS [28], Platanus [29], Allpaths-LG [30], SOAPdenovo 2 [31], and SPAdes [32]. To extract the nuclear genome, blastn (v2.2.30) [33] search against chloroplast and mitochondrial genomes was done in the DB. The nuclear genome was extracted by removing the sequence (mitochondria: 41, chloroplast: 232) and was obtained as a result of a coincidence rate of 99% or more from SC12 _ gapClosed_platanus.fa.

Data of the whole genomes and the gene models of algae, *T. pseudonana*, and *P. tricornutum* were acquired from the Joint Genome Institute project’s home page while those of *V. brassicaformis* was acquired from Ensembl’s homepage. Then, genome size, contigs, the largest contig, total length, GC content, and N50 were evaluated by genome evaluation software, QUAST (v2.3). For the evaluation of repeat sequences, RepeatMasker (version 4.0.6) was used.

For the genome size estimation, we used KmerGenie that could estimate the best k-mer length for genome de novo assembly [34]. KmerGenie also provide estimated genome size using peak k-mer [35].

### Gene prediction, genome annotation and evaluation

Gene prediction and genome annotation were performed by Braker1 software with the default settings (v1.9) [36]. Braker 1 utilized unsupervised training of GeneMark-ET using RNA-seq dataSince Braker utilizes Genome file and Bam file to perform gene prediction, we created a Bam file by applying RNA-seq data to mapping software Tophat. To evaluate gene prediction accuracy, the ECR of the assembly was calculated as the ratio of core genes whose full lengths were expected, including duplicated core genes using BUSCO with Eucaryota odb9 datasets (303 single-copy orthologs) [37].

### Transcriptome analysis

Short read sequences were mapped to the assembled genome using HISAT2 (v2.1) [38] with default settings, and then expression frequencies were calculated using HTseq (v0.10.0) against the estimated gene model (CDS). The differential expression analysis was performed using R based packages, EdgeR (v2.2.0) [39]. The genes identified as differentially expressed genes (DEGs) with FDR < 0.05 were used for further analysis. DEGs were estimated in different sample sets such as different temperatures, different light conditions, and different nutrient conditions.

### List of silicate, Silaffin related genes

Seven genes of silicate and silaffin, Sil1, Sil2, Sil3, SIT1, SIT2, SIT3, and TPSIL2, were extracted from the NCBI DB. Regarding Sil1, we obtained an orthologous gene from *Saccharomyces cerevisiae*. Other genes were obtained from *T. pseudonana*. Then, we performed a blastp search using these seven genes against gene models of *S. costatum*, *T. pseudonana*, *P. tricornutum*, *V. brassicaformis* as a query with the threshold of e-value: 1e - 5.

### List of the harmful algal bloom related genes

Genes and proteins extracted from the paper studying the harmful algal bloom were used as the candidates for the harmful algal bloom causing factors in the diatom ([40, 41], Additional file 1: Table S1, sheet5-HAB genes publication). We classified the information of Gene name, Gene ID, GO categories, and PMID classification. Genes for “Glutamine family amino acid metabolic process,” “Phosphorus metabolic process,” “Response (Phosphorus metabolic process),” “Chloroplast,” “Nitrogen compound transport” in *Arabidopsis thaliana*, and *Chlamydomonas reinhardtii* were selected as the candidate of the harmful algal bloom causal genes. The reason for using *A. thaliana* and *C. reinhardtii* is that they are the model organism for primitive photosynthetic organisms (Additional file 1: Table S1, sheet6-HAB genes in At, Additional file 1: Table S1, sheet7-HAB genes in Cr).

To construct harmful algal bloom gene DB, all genes having homologies to the above categories in *A. thaliana* and *C. reinhardtii* were merged, and the redundant genes were excluded. To find the harmful algal bloom related genes in *S. costatum*, *T. pseudonana*, *P. tricornutum*, we conducted a homology search (blastp) for gene models of these species as well as that of *V. brassicaformis* using harmful algal bloom gene DB with the threshold of e-value: 1e-5.

### Orthologous gene estimation

We estimated the ortholog gene group for *S. costatum*, *T. pseudonana*, *P. tricornutum*, *V. brassicaformis* using Orthofinder (v1.0.7) [42] with default parameters. Orthofinder could find orthologues and orthogroups infers rooted gene trees for all orthogroups and infers a rooted species tree for the species being analysed. Duplicated genes are defined as genes that have more than one gene in the same orthologous gene group defined by orthofinder software.

### Enrichment analysis

We performed Gene Set Enrichment Analysis using DAVID (v6.8, https://david.ncifcrf.gov) on groups of genes extracted in the result section to specify what kind of gene functions were dominant in the groups. To perform the DAVID analysis, ID from *A. thaliana* is necessary as they require GO annotations, so we obtained *A. thaliana* protein sequences and GO annotation file from The Arabidopsis Information Resource (TAIR). *A. thaliana* was chosen as they are most closely related to diatoms. Next, we conducted homology search for the genes involved in the groups extracted in the result section against the blastdb of *A. thaliana* proteins. *A. thaliana* ID was then converted to RefSeq protein ID using BioMart to perform gene enrichment analysis by DAVID.

### Pathway analysis of oxidative phosphorylation

The pathway of oxidative stress response and response to cytokinin were taken from the KEGG database. Genes involved in these pathways were extracted, and gene expression intensities (FPKM) of these genes were used for heatmap analysis.

## Additional file

Additional file 1: **Table S1.** Sheet-1, The number and length (bp) of each repetitive element in four species, Sheet-2, The number of genes from Harmful algal bloom related genes, selected as shown in the methods, Sheet-3, Highly duplicated genes in Sc, and their annotations. Kog representes functional ID of eukaryotic cluster of genes, Sheet-4, The number of silicate and silaffin related genes in four species, Sheet-5, Genes known to be related to harmful algal blooms in the publications, Sheet-6, Orthologs of HAB genes found in *Arabidopsis thaliana*, and used in this study, Sheet-7, Orthologs of HAB genes found in Clamydomonas reinhardtii, and used in this study. (XLSX 502 kb)

## Abbreviations

CDS: Coding sequences
DB: Database
ECR: Eukaryotic common gene conservation rate
GO: Gene ontology
HAB: Harmful algal bloom
SIT: Silicon transporter
